# Warfare-induced mammal population declines in Southwestern Africa are mediated by species life history, habitat type and hunter preferences

**DOI:** 10.1038/s41598-020-71501-0

**Published:** 2020-09-17

**Authors:** Franciany Braga-Pereira, Carlos A. Peres, João Vitor Campos-Silva, Carmen Van-Dúnem Santos, Rômulo Romeu Nóbrega Alves

**Affiliations:** 1 Department of Ecology and Systematics, Universidade Federal da Paraíba, João Pessoa, PB 58051-900 Brazil; 2 School of Environmental Sciences, University of East Anglia, Norwich Research Park, Norwich, NR4 7TJ UK; 3 Faculty of Environmental Sciences and Natural Resource Management, Norwegian University of Life Science, Ås, Norway; 4 Institute of Biological and Health Sciences, Universidade Federal de Alagoas, Maceió, AL 57072-900 Brazil; 5 Department of Biology, Universidade Agostinho Neto, Avenue 4 de Fevereiro, 000000 Luanda, Angola; 6 Laboratory of Ethnobiology and Ethnoecology, Universidade Estadual da Paraíba, Campina Grande, 58429-500 Brazil

**Keywords:** Ecology, Zoology, Environmental sciences, Environmental social sciences

## Abstract

Civil wars often coincide with global biodiversity hotspots and have plagued the everyday reality of many countries throughout human history. However, how do civil wars affect wildlife populations? Are these impacts the same in savannah and forest environments? How persistent are the post-war consequences on wildlife populations within and outside conflict zones? Long-term monitoring programs in war zones, which could answer these questions, are virtually nonexistent, not least due to the risks researchers are exposed to. In this context, only a few methodologies can provide data on wild populations during war conflicts. We used local ecological knowledge to assess the main consequences of a prolonged civil war (1975–2002) in Southwestern Africa on forest and savannah mammals. The post-war abundance in 20 of 26 (77%) mammal species considered in this study was lower in open savannah compared to the closed-canopy forest environments, with some species experiencing a decline of up to 80% of their pre-war baseline abundance. Large-bodied mammals were preferred targets and had been overhunted, but as their populations became increasingly depleted, the size structure of prey species gradually shifted towards smaller-bodied species. Finally, we present a general flow diagram of how civil wars in low-governance countries can have both positive and negative impacts on native wildlife populations at different scales of space and time.

## Introduction

Many developed and developing countries have succumbed to prolonged civil wars and other armed conflicts throughout modern human history^1^, leaving more than 30 million people displaced in the last 20 years alone^2,3^.Contemporary wars often result in politically inaccessible areas for resource users, particularly where land mines are widely and unpredictably scattered, severely discouraging human settlements and game hunters, thereby creating potential game refuges as passive ‘no-take’ areas^4–6^. However, armed conflicts can induce dramatic direct and indirect impacts on wildlife populations and natural ecosystems^7^. Civil wars can dismantle the ‘law-and-order’ structure of conservation institutions and vastly increase the availability of automatic weapons and ammunition, which can be used by residents, political refugees and military troops to deplete wild game for subsistence and trade in both military and peri-military areas^1,8,9^.

Armed conflicts are widely distributed in Africa, having occurred in 71% of all Afrotropical Protected Areas and over 80% of biodiversity hotspot areas between 1946 and 2010^9,10^. Recurrent episodes of military violence have coincided with dramatic changes in wild populations of 69 African mammal species > 5 kg, which declined by 59% between 1970 and 2005^11^. In addition, elephant (*Loxodonta africana*) and hippopotamus (*Hippopotamus amphibius)* populations in Virunga National Park (DRC) have been reduced by up to 95% because of unregulated hunting practices using automatic weapons^12^. In addition, changes in socio-economic context can markedly reduce the financial and human resource allocation for nature conservation, thereby becoming even more pervasive than the tactical consequences of armed conflicts^13^.

During the years of warfare conflict, large mammals are particularly vulnerable to sudden changes in hunting pressure due to their high subsistence and commercial value in terms of meat and other body parts. In contrast, birds, fish, reptiles, amphibians, and invertebrates are more likely to be indirectly affected through warfare-mediated changes in habitat conversion^13^. Species body mass markedly influence the choice of game species by hunters, but other factors such as local species abundance and food taboos can also play an important role^14^. Vegetation types and landscape structure can also influence the dynamics of game pursuit. Wildlife in open-habitat areas, such as sparsely wooded savannahs, can increase the intrinsic detectability of desirable target species, exposing them to higher mortality induced by hunters in vehicles or on foot^15^.

**Figure 1:**
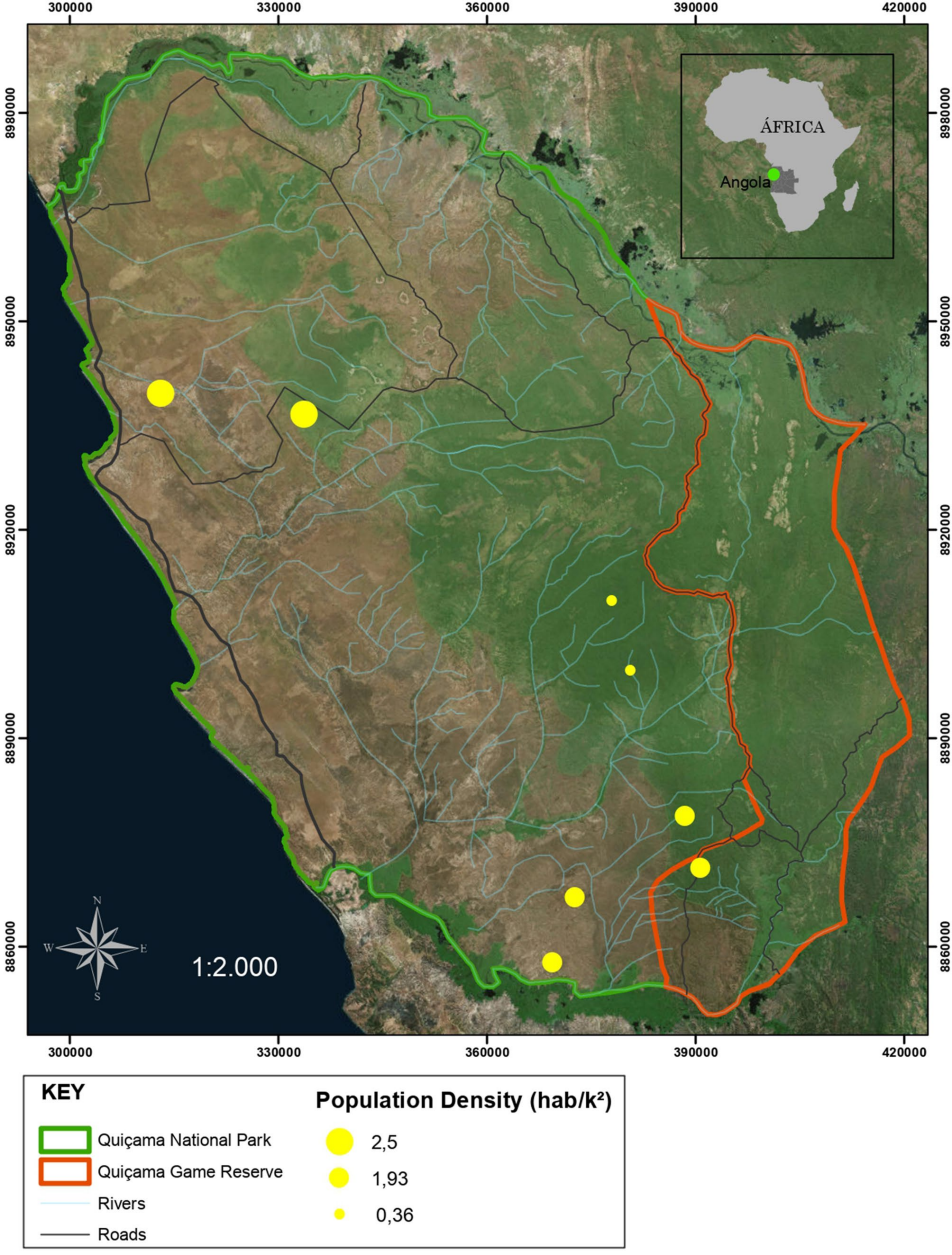
Location of Quiçama National Park (demarcated by the green line) and Quiçama Game Reserve (demarcated by the orange line) in West Africa. Solid yellow dots indicate the surveyed townships, which encompassed more than one human settlement. Dot sizes are proportional to the population size of the townships. Green and greyish –orange background areas represent forest and savannah environments, respectively. Map generated using ArcGIS 10.3.1; Datum: WGS84 Source: ESRI, Edited in Adobe Photoshop and Elaborated by Ana Caroline Imbelloni; Franciany Braga-Pereira in January/2019.

The rehabilitation or recovery of mammal populations ideally requires an understanding of pre-disturbance baselines and broad assessments of the environmental effects of civil wars. This information is imperative to facilitate mitigation plans in conflict-prone regions, so there is enormous value in characterizing multi-decadal wild population mass-mortality events. Angola serves as an excellent example of how a prolonged civil war can affect wild mammals populations. The country is home to at least 275 species of mammals^16^, many of them historically hunted by the local communities as a source of protein before, during and after the intermittent 27-year of Angolan civil war^17^, started after the independence from Portugal in 1975^18^. Here, we conduct the first chronological-scale analysis (pre-, during, and post-war periods) of the effects of the prolonged Angolan civil war (1975–2002) on the abundance status of terrestrial non-volant mammal populations larger than 1 kg in two major West African protected areas in both forest and savannah environments (Fig. 1). We assessed data derived from Local Ecological Knowledge (LEK), retrieving past information and projecting future trends. In the 1970s, populations of elephant (*Loxodonta africana*), forest buffalo (*Syncerus caffer nanus*), eland (*Tragelaphus oryx livingstonii*) and roan antilope (*Hypotragus equinos cottoni*) in the study area were consistently present and widespread^19–21^. However, we show that direct and indirect impacts of this civil war facilitated game overexploitation, inducing rapid population collapse of these large-bodied mammals. We also found marked changes in the abundance of residual assemblages of game species, given that prey assemblages gradually shifted towards smaller-bodied species, mainly in savannah landscapes. Finally, we present a model of how civil wars in low-governance countries can have both positive and negative impacts on native wildlife, through socio-political changes, in detriment of national economies and their natural resource capital.

## Results

### Civil war influence mechanisms on wild mammals

Consistent with local perception, we found that the main impacts of the Angolan civil war on wildlife populations of the Quiçama region were indirect, ultimately arising from institutional and socio-economic changes, rather than from direct military tactics. These included the widespread distribution of automatic rifles and ammunition (stated in 100% of informant responses); suspension of park budgets to fund anti-poaching patrols (70%); installation of military bases within core conservation areas (69%); over-hunting and mammals migration during and after the war (68%); and new settlements of displaced refugees in the northern portion of QNP, as a direct consequence of armed conflict intensification in Eastern Angola (25%), all of which strongly impacted game species (Supplementary Information, Fig. A1).

Our qualitative results in relation to game offtakes before, during and after the war, the causal mechanisms for local abundance shifts and declines in target species, and future prospects for wildlife at Quiçama are available in Supplementary Information, S1.

### Species distribution and abundance changes

The terrestrial mammal assemblage > 1 kg of Quiçama National Park and Quiçama Game Reserve was comprised of at least 32 native species (Table 1). Of those 32 species, local hunters were confident to estimate the abundance for all pre- and post-war periods of 26 hunted species. Four mammal taxa explicitly mentioned in the interviews could only be identified to the level of genus or family, rather than species.

**Table 1:** Nonvolant terrestrial game mammal species larger than 1 kg recorded within Quiçama National Park (QNP) and Quiçama Game Reserve (QGR), Angola.

Order	Binomial name		English name	Body mass (Kg)	Annual fecundity rate	IUCN Red List Categories	Habitat
Proboscidea		*Loxodonta *sp.	Elephant	3,824	0.168	VU	F/S
Artiodactyla		*Syncerus caffer nanus*	Red Buffalo	592.7	0.432	VU	F/S
Artiodactyla		*Tragelaphus oryx*	Eland	562.6	1.14	LC	S
Artiodactyla		*Hippotragus equinus*	Roan Antelope	264.2	1.1564	LC	S
Carnivora		*Panthera leo*	Lion	158.6	1.375	VU	F/S
Artiodactyla		*Hippopotamus amphibius capensis*	Hippopotamus	1536	0.6	VU	F/S
Artiodactyla		*Potamochoerus larvatus*	Bushpig	69.1	3.003	LC	F/S
Artiodactyla		*Phacochoerus aethiopicus*	Warthog	75.6	3.2	LC	S
Carnivora		*Crocuta crocuta*	Hyena	63.4	1.91	LC	F/S
Tubulidentata		*Orycteropus afer*	Aardvark	56.2	1.1	LC	F/S
Carnivora		*Panthera pardus*	African Leopard	52.4	1.6264	VU	F/S
Artiodactyla		*Redunca arundinum*	Common Reedbuck	70.0	1.2446	LC	S
Artiodactyla		*Tragelaphus scriptus*	Bushbuck Kewel	50.0	1.37	LC	F/S
Artiodactyla		*Sylvicapra grimmia*	Common Duiker	15.6	1.96	LC	S
Rodentia		*Hystrix africaeaustralis*	Cape Porcupine	14.9	2.265	LC	F/S
Carnivora		*Lycaon pictus*	Wild-dog	22.0	8.991	EN	F/S
Carnivora		*Civettictis civetta*	African Civet	12.1	3.3264	LC	F/S
Carnivora		*Leptailurus serval*	Serval	12.0	5.875	LC	F/S
Carnivora		*Mellivora capensis*	Honey badger	8.9	2.35	LC	F/S
Carnivora		*Canis adustus*	Side-striped Jackal	8.2	4.9403	LC	F/S
Artiodactyla		*Philantomba monticola*	Blue Duiker	6.0	1.2642	LC	F/S
Primates		*Cercopithecus mitis*	Blue Monkey	5.0	0.88	LC	F/S
Carnivora		*Felis silvestris cafer*	African wild cat	4.5	3.5	LC	F/S
Rodentia		*Thryonomys swinderianus*	Marsh Cane Rat	4.0	3.3	LC	F/S
Lagomorpha		*Lepus victoriae*	African Savannah Hare	1.5	8.58		F/S
Primates		*Chlorocebus cynosurus*	Vervet Malbrouck Monkey	5.0	1	LC	F/S
Hyracoidea		Hyracoidea	Hyrax	~ 2.8		LC	F/S
Carnivora		Herpestidae	Mongooses			LC	F/S
Carnivora		*Genetta maculata*	Blotched Genet	1.8	3.5724	LC	F/S
Primates		*Miopithecus talapoin*	Talapoin	1.2	1.111	LC	F/S
Primates		*Galago crassicaudatus*	Thick-tailed Galago	1.2	1.254	LC	F/S

Species conservation status based on the most recent red-list from the International Union for Conservation of Nature (2019.2): *LC* least concern; *VU* vulnerable; *EN* Endangered. Habitat type: *F* Forest; *S* Savannah. Silhouette credits: F. Braga-Pereira.

**Figure 2:**
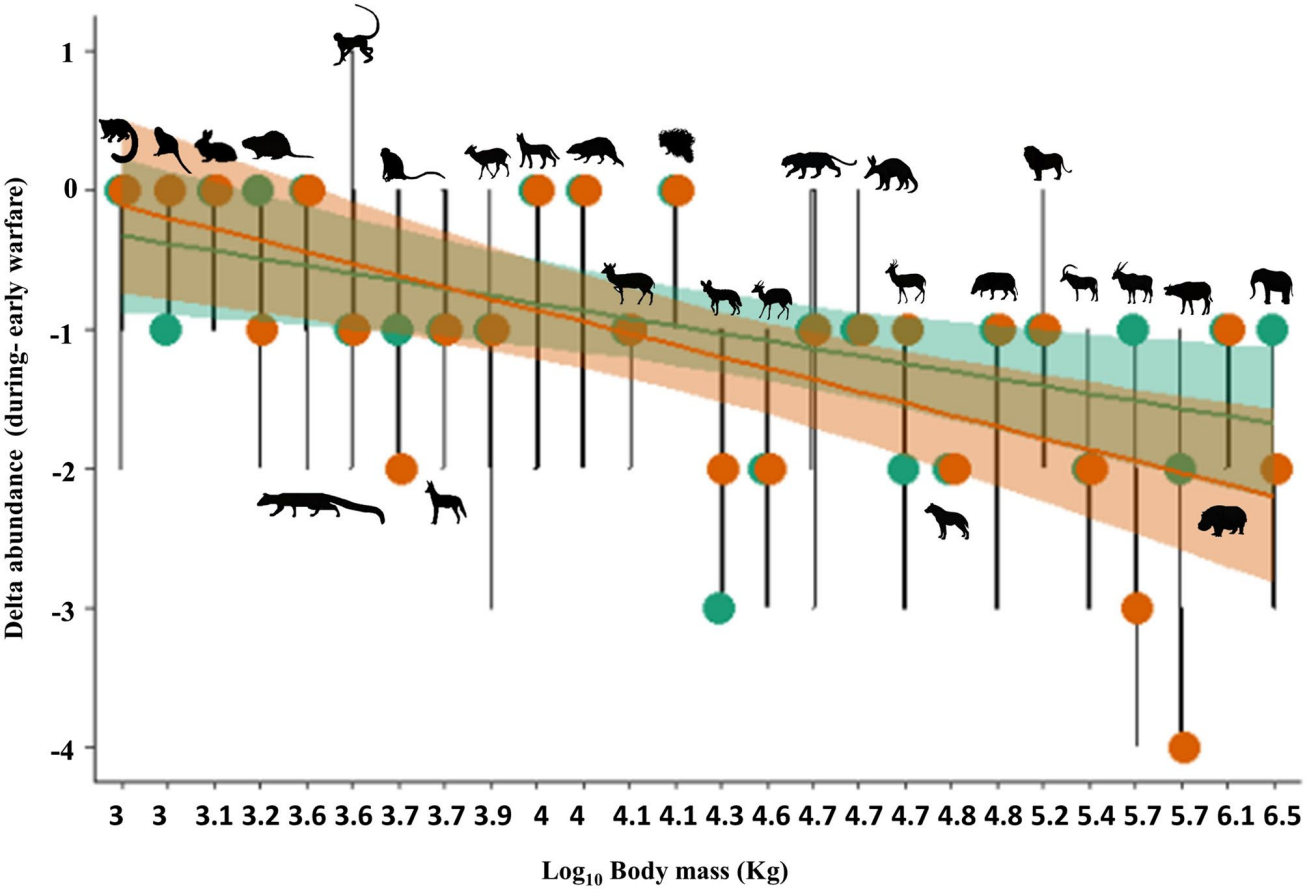
Differences in mammal species relative abundance (∆_ab_) before and after the armed conflict in Angola as a linear function of species body mass (log x). Orange and green solid dots (and 95% confidence regions) represent values for savannah and forest environments, respectively. The beta- coefficient value is 0.2298 and the R^2^ 0.4075.

Six of all identified species occurred mainly in open savannah landscapes, and only occasionally ventured into closed-canopy forest: eland, roan antelope, common reedbuck*,* common duiker*,* warthog, and honey badger. Comparing our metrics of wild mammal abundance before and during the war, we found that populations of 20 of the 26 mammal species (77%) assessed were reduced during the war, particularly large-bodied species in open-savannah environments (Fig. 2). Population depletion was clearly related to species body size, with all six of the smallest-bodied species (vervet malbrouck monkey, Genet, african savannah hare, talapoin, thick-tailed galago) showing stable populations during the war. For example, in terms of the 4-point ∆_ab_ scale, elephants succumbed to a population reduction of 2 points in savannah and 1.5 in forest environments, whereas rabbits experienced no perceived decline in both landscapes.

Considering each time interval for each species, none of the species were ranked as either “absent” or “very low” abundance prior to the war. The median abundance value of all large and medium-sized species (except for cape porcupine) and some small-bodied species (blue duiker and blue monkey) in savannah areas declined during the war and did not recover during the post-war period.

**Figure 3:**
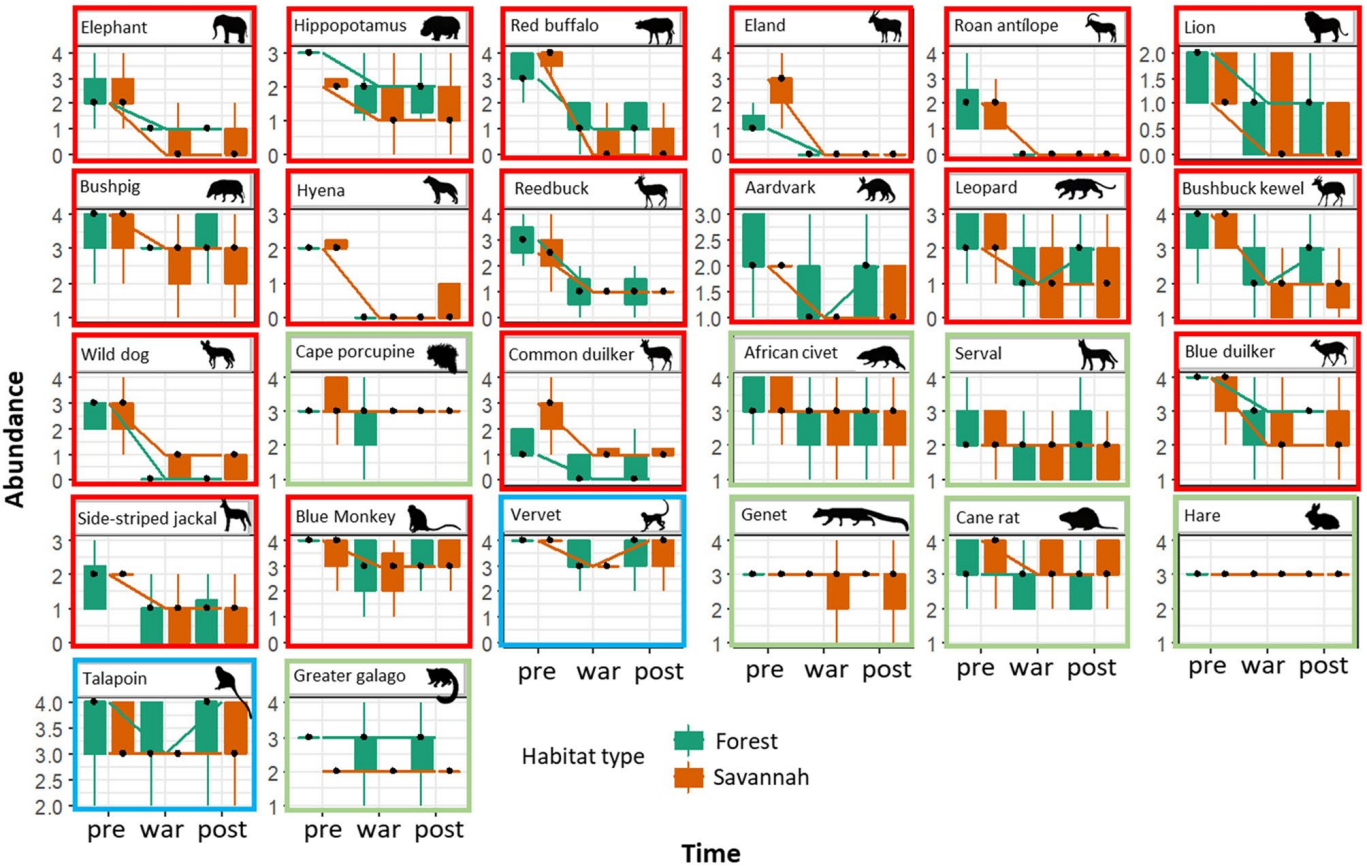
Relative abundance of species during Angolan pre-war, war and post-war periods. Orange and green boxes represent the abundance in savannah and forest environments, respectively. Solid black dots represent median values. Perceived relative abundance is on a scale ranging from 0 (when the species was *absent*) to 4 (when the species was *highly abundant*).

In contrast, in forest landscapes, 15.8% of all large and medium-sized species (aardvark, leopard and bushbuck kewel), whose abundance had declined during the war, have since experienced post-war population increases (see red-highlighted panels in Fig. 3). In both landscapes, 46% of all medium- and small-bodied species experienced no changes in their median relative abundances during all periods assessed (see green-highlighted panels in Fig. 3). Three primate species (blue panels in Fig. 3) showed a decline in their abundance during the war but have since recovered during the post-war period. Median abundance values indicate that some large-bodied species were still missing during the post-war period, as few informants had mentioned seeing or capturing these species during this period. In this case, we recorded only one lion capture in 2012 at Omba, two captures of eland in 2010 in the boundary between Cacharandanda and Mumbondo, one capture of wild dog in 2014 at São Braz and tree sightings of hyena in 2016 at Bravo 2. For elephant and red buffalo, in addition to evidence from informants we have records of dung and spoor for several areas in the southern portion of Quiçama, for both savannah and forest landscapes (Supplementary Information, Fig. 2).

### Target species

**Figure 4:**
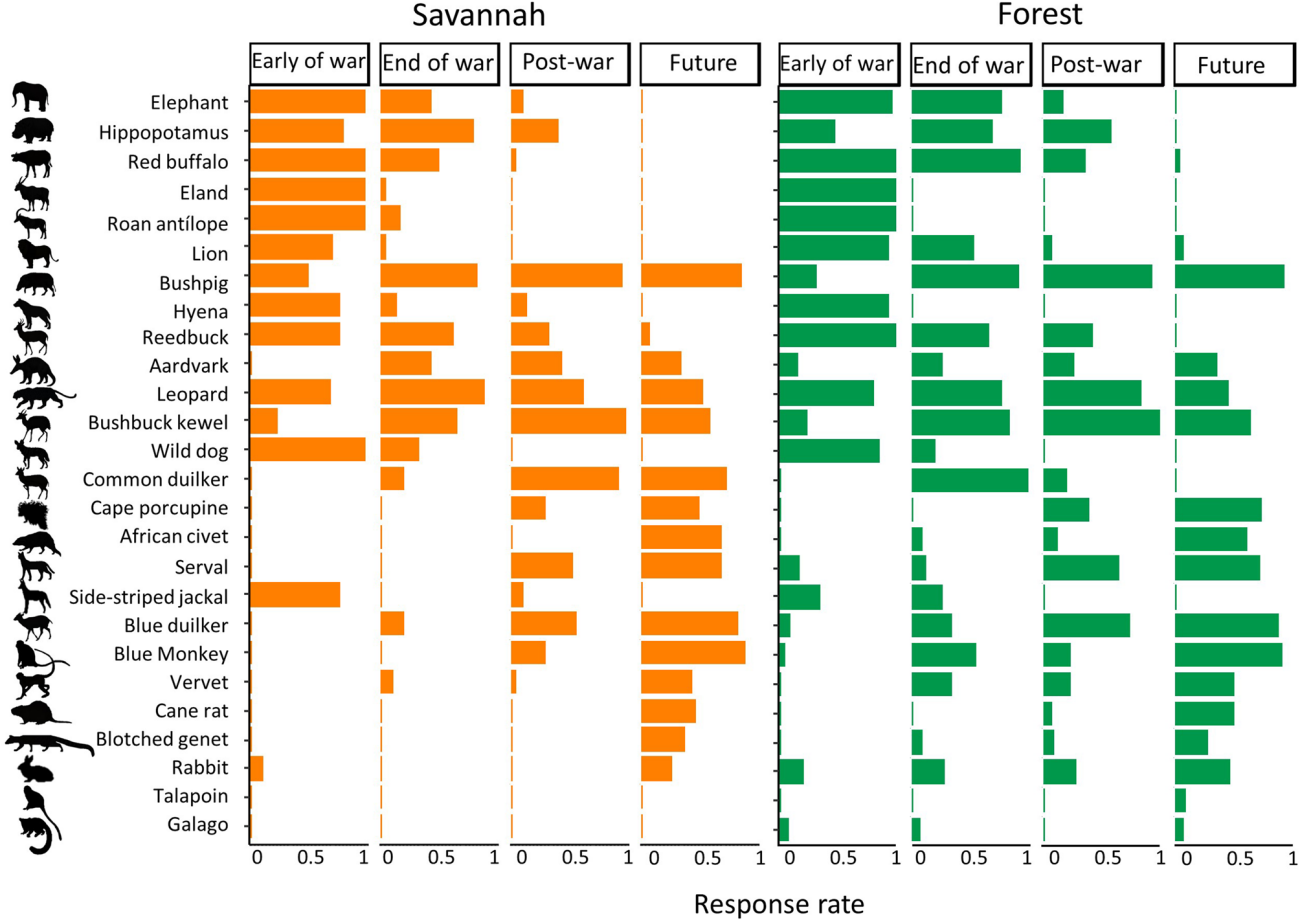
Percentage of responses from local hunters related to each potential game species in terms of whether or not they were actually hunted as a target species during each of the four assessed periods before and after the Angolan armed conflict—early (1975 to 1988) and late war periods (1989 to 2002), present (2003 to 2017), and future (post 2019). War 1 and War 2 indicate the first and last half of the war period (years), respectively.

We identified as game targets those species that are commercially valuable and could be sold as bushmeat or other body parts, or species from which hunters obtained high rates of wildmeat return and were therefore consistently pursued. Early in the war, target species were comprised primarily of medium- to large-bodied species, but over time as the armed conflict intensified and spread geographically hunters pursued primarily small to medium-bodied species. During the late stages of the war, hunters in savannah environments largely pursued and killed medium-sized species. In contrast, hunters operating in forest areas continued to kill medium- and large-bodied species until the late stages of the war, and were still pursuing a larger number of species compared to hunters in savannah areas. After the war, hunters largely pursued medium-bodied species in both savannah and forest landscapes, further indicating that large-bodied prey had been depleted. Finally, hunters reported that, as a future cognitive projection, they would be restricted to hunting only small-bodied species such as Rabbit and Greater Cane Rat, including species that had not been reported as desirable targets during any of the present and past time periods (Fig. 4).

### Predictors of prey abundance and target species composition

**Figure 5:**
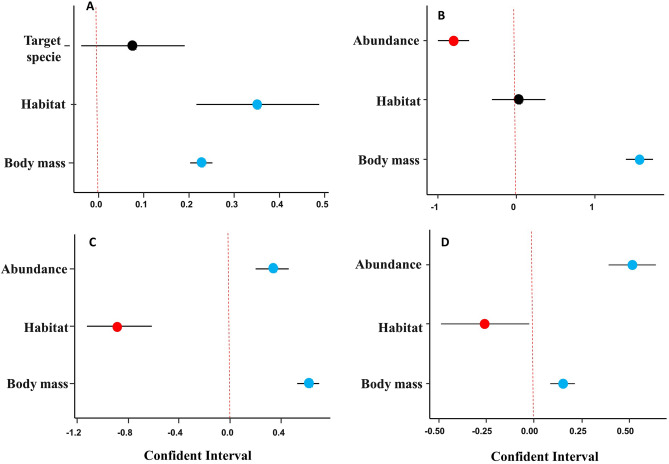
Linear coefficient estimates (± 95% confidence intervals) showing the magnitude and direction of effects on differences in reported population abundance in the Quiçama region of Angola between the pre-war and post-war years (**A**). Effects of different variables on the choice of target species during the early-war years (**B**), late war years (**C**) and during the aftermath of the war (**D**). Blue and red solid dots represent either significantly positive or significantly negative effects, respectively.

Models explaining changes in abundance revealed a significant decline in the abundance of large-bodied mammals, mainly in savannah areas compared to forest environments. Whether or not a species was preferentially hunted, in itself, did not have a significant effect on warfare-mediated population depletion (Fig. 5A). In relation to which species were reported as hunting targets, the least abundant species were hunted during the early war period (Fig. 5B), but hunting targets shifted to the most abundant prey species during the late and post-war periods (Fig. 5C,D). Savannah environments did not show an overall difference in the total number of species reported as targets at the onset of the war (Fig. 5B), but this number had become significantly lower by the late and post-war periods compared to forest habitats (Fig. 5C,D). Our data also show that larger-bodied species were the most frequently pursued prey by hunters during all periods assessed (Fig. 5B–D). We found that mammal body size and perceived abundance had significant effects on which species were hunted during the early war period, but these effects also included habitat type (forest or savannah) during and after the war.

## Discussion

Compared to the pre-war baseline, our results show an overall numerical population depletion of 77% across all mammal species during the war period, with some species experiencing a decline of up to 80% of their pre-war baseline abundance. Moreover, this degree of wildlife decline was not reversed by the end of the post-war period. This overall pattern of marked large mammal declines has not been previously documented at sites exposed to intense armed conflicts, which in Angola and other combatant countries profoundly dismantle the socio-political structure, natural resource management activities and enforcement practices such as bushmeat market inspection^22,23^. We emphasize that even during post-war peace times, wild mammal populations in Angola will fail to recover as long as rural people living in war-torn countries remain armed and wildlife management regulations cannot be enforced.

In Angola, there has been a process of slow disarmament of citizens by the government, which has disrupted hunting practices and reduced hunting pressure on local wildlife populations. However, meaningful recovery of institutional policy on protected areas and wildlife populations have not yet been implemented in all the Quiçama region, which is now largely occupied by a mix of native peoples, war refugees, and former combatants. As a consequence, post-war mammal population rebounds have been entirely restricted to some small-bodied species, likely due to their higher fecundity, in contrast with the low reproductive rate of medium- to large-bodied species, which continue to be slaughtered by fire weapons and other hunting techniques. Automatic rifle confiscation from citizens is an important factor in reducing hunting pressure, thereby favouring the recovery of local game biomass^13,24^. However, without the critical intervention of well-designed government policies, the baseline structure of large terrestrial vertebrate assemblages is unlikely to recover. For example, in the post war-zone Gorongosa National Park, Mozambique, the total biomass density of nine focal large mammal species had recovered in 2018 by ~ 80% of the pre-war baseline density, but the community composition had shifted dramatically compared to the pre-war baseline due to asymmetric recovery rates across species, with smaller antelope species exceeding the abundance of formerly dominant megaherbivores^25^. In particular, waterbuck abundance had increased by an order of magnitude, with more than 55,000 individuals accounting for over 74% of large-herbivore biomass by 2018. By contrast, elephant, hippo, and buffalo, which accounted for 89% of the pre-war biomass, now comprised only 23%^25^.

Considering carnivores, only lion populations in Mozambique’s Gorongosa National Park persisted throughout the war^26^, whereas leopards also persisted at intermediate abundance in forest environments in our study area. Both of these studies also recorded hyenas and jackals. At Quiçama, however, only two local informants had seen or killed hyenas or jackals over the last 5 years. The collapse of these carnivores has important ecological implications on their roles in key ecosystem linkages, such as necromass scavengers and energy and nutrient transfer^27^.

Defaunation can have important impacts not only in terms of severe depletion of vulnerable species but also on general ecosystem functions, including predation, herbivory, carrion removal and disease control^28,29^. For example, the Mozambican Civil War (1977–1992) induced to a catastrophic large‐herbivore die-off in Gorongosa National Park, which was followed by 35 years of woodland expansion, most severely in areas where pre‐war herbivore biomass was greatest^7^. This expansion included the invasive *Mimosa pigra* shrub—considered one of the world’s 100 worst invasive plant species^30^. Tree cover increased in four of the park's five major habitat zones by 51% to 134%. Local informants in our study explained that in many areas of Quiçama the landscape have become more wooded since the collapsed of large herbivores, although this remains anecdotal. The most parsimonious explanation in both Mozambique and Quiçama is that a severe reduction in browsing pressure enhanced tree growth, survival and/or recruitment^7^.

Before the Angolan civil war, the protected areas of the Quiçama region once safeguarded one of the largest world populations of Red Buffalos (around 8,000 individuals) across both savannah and forest landscapes^31^. However, we found that poaching had severely reduced Red Buffalos to small populations restricted to some forest fragments in the southern Quiçama area. Landscape structure and vegetation cover clearly interfere with the degree of hunting efficiency because they affect hunter velocity, understorey visibility, size-selective prey detectability, and hunting techniques. In open savannah areas, larger animals can be easily detected, resulting in far more efficient use of long-range projectiles fired by automatic rifles and other weapons carried by distant hunters^17^. Also, compared to forest environments, motor vehicles gain much more feasible access into savannah landscapes when both pursuing prey and transporting carcasses to markets, which further explains the higher depletion rates of the savannah megafauna^32^. Mammals inhabiting more accessible open areas are therefore more vulnerable. For example, a study on Europe's largest terrestrial mammal (*Bison bonasus*) showed that stronger pre-historic hunting pressure in open landscapes forced these animals into closed-canopy forest as a refuge habitat since the Pleistocene, leaving the legacy of the last native bison populations being restricted to forest areas^15^. However, habitat quality in forest refugia is not necessarily suitable. For instance, eland and roan antelope at Quiçama were unable to seek refugia in forest remnants, unlike other large-bodied species such as elephant and red buffalo. This likely explains why over 90% of our interviewees reported the conspicuous absence of those two ungulate species in the entire area.

Our model shows that commercially valuable target species in both savannah and forest habitats were not necessarily the most abundant during the early stages of the war. This is likely because the abundance of large-bodied species was then not low enough to discourage hunters from pursuing them. However, during the late and post-war periods, depletion rates of large-bodied prey in savannahs habitats were so high that pursuing them had become less worthwhile than pursuing midsized species. Because of the elevated time/energy costs of capturing large-bodied prey species in savannah areas, hunters become more selective in this habitat compared to the forest. On the other hand, given that levels of depletion of large-bodied species in forest areas were lower, most of these species continued to be killed in this habitat type, but resulted in smaller offtakes. Hunters also selected midsized species to compensate for any losses in the overall biomass of prey profiles. In the aftermath of the war, the gradual shift in prey size structure towards smaller-bodied species progressed and midsized species were most frequently selected by hunters in both savannah and forest habitats. In a study in Ghana, commercial trophy hunting for ivory, as opposed to subsistence hunting, was more sensitive to the density of elephants and enforcement efforts to inhibit poaching, supporting the notion that commercial hunting often depends mainly on overall prey abundance^33^.

Hunter preference for large- and medium-bodied species is higher because they yield higher catch-per-unit-effort in terms of meat biomass and other products (e.g. ivory and skin). As such, most species smaller than 12 kg were not a target game species and their relative abundance remained unchanged over the assessed periods. The fact of whether or not any given species had been reported as a hunting target during the war did not affect its pre- to post-war change in perceived abundance (see Fig. 4A) was influenced by the depletion of some small-bodied species which were not commercially harvested during the war, but were still hunted—because they were crop-raiders or depredated livestock—at a time when plenty of ammunition was readily available. That subsistence and/or commercial game hunting can have a profound detrimental effect on the biomass of large-bodied species has been widely documented^34,35^. However, we note that the abundance of medium-sized species at Quiçama continues to decline. In contemporary Africa, mammal populations have shown a ‘U-shaped’ abundance trend. Perhaps because small-bodied species are higher-fecundity and/or bypassed by hunters, large-bodied species have been targeted by wildlife management and conservation programs, whereas intermediate-sized species have experienced the steepest declines as they are usually hunted, but lack active management and can exhibit slow reproductive rates^36^. Therefore, there is a need to also directly manage midsized species, rather than assume that management actions targeting the most iconic ‘umbrella’ taxa will lead to effective conservation of all species. In our study area, for example, the greatest conservation focus should be allocated to bushbuck (*Tragelaphus scriptus*), currently the most hunted species at Quiçama (mainly for trade). This ungulate species has received no attention from regional to national scale conservation programs^37^.

We found little or no change in the relative abundance of small mammals, perhaps because these small-bodied species were neither commercially valuable nor harvested for local subsistence. However, comparing our results with other studies using combined sampling techniques such as camera traps, net, and microphones^16^, we recognize that some small mammals could have been undersampled, despite the enormous usefulness of LEK approaches in meeting the aims of this study. Regarding the primates, cultural influences such as food taboos may have important roles in mediating population declines of overexploited species. However, primates elsewhere in Africa and the Neotropics comprise the largest number of species threatened by hunting across the world’s mammals^38^. We therefore caution that the future bushmeat trade in Angola could, in fact, begin to target primates as other more desirable large-bodied species become gradually depleted and economically extinct. In addition, we highlight the increased risk of zoonotic diseases, given that our close phylogenetic relationship with nonhuman primates increases the likelihood of animal-to-human pathogen spillover^39^ and because the risk of disease emergence among mammalian orders is highest in bats (risk rate = 2.64), followed by primates (2.23), ungulates (2.09), rodents (1.81) and carnivores (1.39)^40^.

Modern armed conflicts affect terrestrial wildlife through a range of interactions, including tactical military operations. However, the consequences of socio-economic upheaval and livelihood disruption associated with a civil war can outweigh the direct effects of military activity^9^. Among the 24 mechanisms through which armed conflicts are known to affect wildlife, eight (86% of all existing case studies by 2016) were "non-tactical" pathways involving institutional decay, displacement of people and economic upheaval^13^. Accordingly, our results show that the main consequences of the war in the Quiçama region were non-tactical, such as much greater access to powerful fire-weapons, which were widely used by hunters and the military, even though their initial distribution purpose was to arm the population to fight against rival militias. The widespread use of automatic weapons intensified the overkill of large mammals, increasing hunting efficiency and the number of hunted species. In addition, wildlife culls were intensified during all brief periods of cease-fire because once the probability of encountering guerrilla groups was reduced, armed hunters felt safer and increased the amount of time allocated to hunting activities as well as the size of their catchment areas.

Ivory tusks from elephants killed at Quiçama were removed by the natural resource sector of each political party responsible for the catch, probably in exchange for automatic weapons^1,41^. Consequently, Angola’s elephants during the 1980s drew international alarm with reports of up to 100,000 elephants exterminated within rebel-controlled territories^42^. Park rangers were also victims of the threat from rebel groups, which was exacerbated by hundreds of outside hunters gaining access to the Quiçama area. Similarly, in the Okapi Reserve in the Democratic Republic of Congo, park guards were forced to abandon their posts following guerrilla attacks and were unable to prevent elephant poaching and bushmeat extraction^13,43^.

Strategic installation of both fixed and mobile military bases throughout protected areas is a tactical manoeuvre that greatly facilitates access to rifles and ammunition by all residents. However, in some situations this can potentially benefit wildlife populations elsewhere by effectively creating a “no human’s land”. This was the case in the Demilitarized Zone separating North and South Korea, which has been uninhabited by humans, thereby becoming a unique nature reserve containing the last refugia of Korean natural heritage^23^. Therefore, some pathways can show both positive and negative consequences for wildlife, depending on the spatial extent and timescale considered. In fact, if on one hand, exclusion zones often create protected areas for wild nature, on the other hand, sites overrun by war refugees will succumb to much greater hunting pressure. Where the civil war was most intensive in Eastern Angola, many populations of endangered wild species have been identified^44^, whereas in Western Angola, where the armed conflict was patchy or episodic, we found that wild populations of a similar set of species spiralled down into steep declines or were driven to local extinction. Despite intensive post-war efforts in clearing and deactivating landmines, millions of hectares of these explosive weapons zones remain under interdiction in Europe, Africa, and Asia^45^. This unpredictable distribution of landmines is also a double-edged sword because many refugees did not return to their original households after the war terminated because of risks associated with landmines. Some of the most intact ecosystems of Central America, for example, have not been threatened by habitat conversion by agrarian peasants because they were seeded with landmines during the civil wars^46^. Nevertheless, landmines also pose threats to wildlife, killing for example at least 30 elephants in Angola’s southern provinces^42^. Also, when landmines explode, they shatter soil systems, rip up plant life and disrupt water flows, all of which accelerate widespread ecosystem disruption^46^.

**Figure 6:**
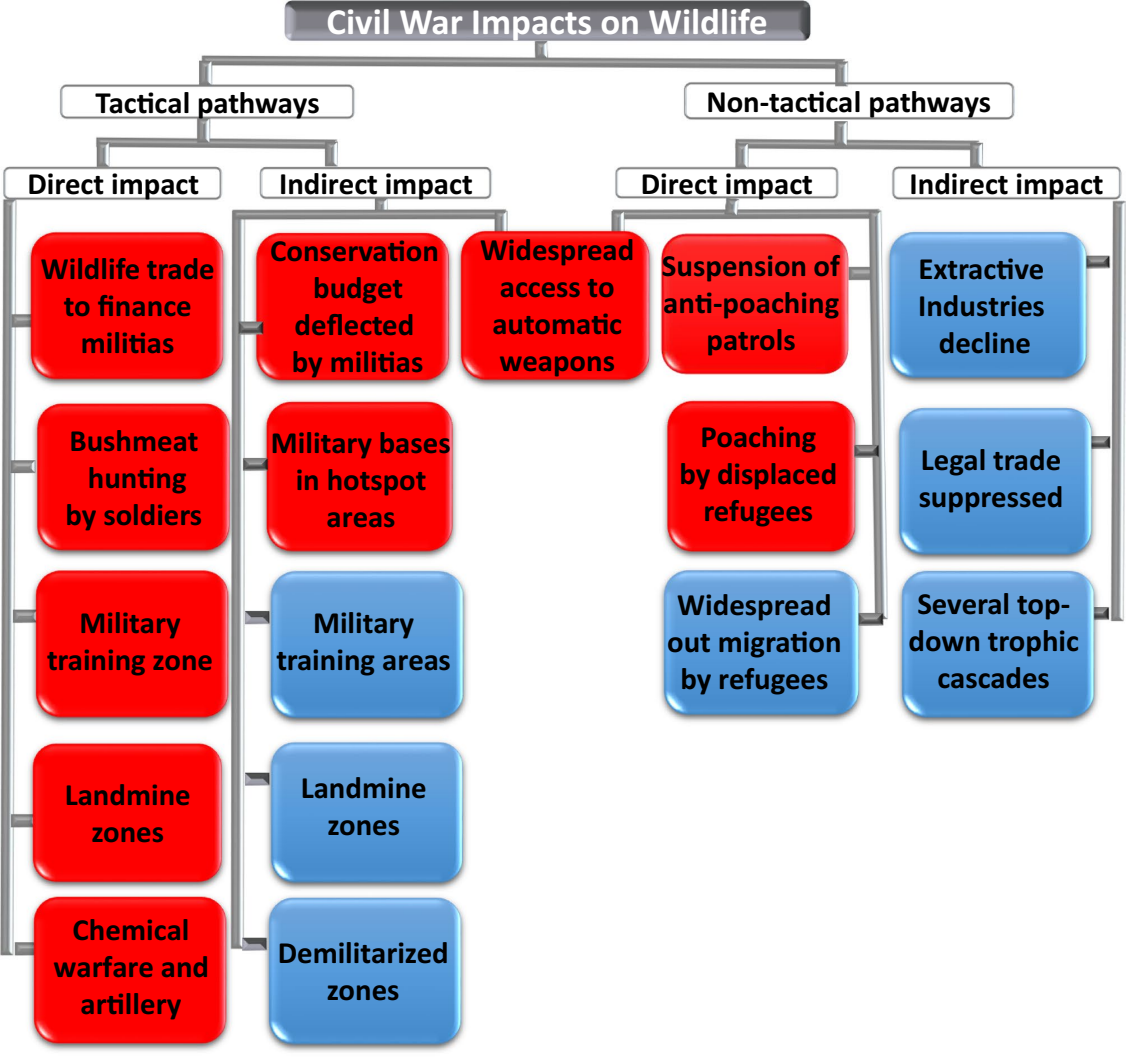
Pathways through which modern armed conflicts can affect wildlife populations within war zones. Distinct pathways linking armed conflict to wildlife outcomes organized thematically in “tactical” pathways (which arise directly from the conflict itself and are associated with military tactics or supporting military activities) and “non- tactical” pathways (which stem from broad socio-political and economic changes associated with the armed conflict, including changing institutional dynamics, movement of people, and altered economies and livelihoods). Blue and red boxes represent either positive or negative effects, respectively.

The main impacts of the Angolan civil war on terrestrial mammals of Quiçama occurred indirectly from military tactics or from “non-tactical” pathways and resulted from wholesale institutional and socioeconomic changes, rather than directly from military tactics. In view of all our findings and related literature, we present a summary flow diagram showing how modern armed conflicts can impact wildlife in modern war zones (Fig. 6). We divide the impact of wars into (A) tactical pathways, which are directly or indirectly derived from military unrest, associated military tactics or supporting military activities; and (B) “non-tactical” pathways, which stem from broad socio-political and economic changes associated with armed conflicts, including major institutional or policy failure, movement of refugees, and severely altered economies, local livelihoods and ecosystems.

Finally, we highlight that 36 countries worldwide are currently experiencing civil wars and most of these conflicts are fuelled or funded by international interests or started after an external intervention. These internationalized conflicts are more prolonged and less likely to find a political solution^47^. Mirroring our study area, protected areas confronting military conflicts elsewhere become surrounded by armed citizens and can rely on little, if any, national and international support to combat poaching by armed people^48^. Therefore, considering measures can reduce the impact of warfare on wildlife, we emphasize the intentional or inadvertent complicity of foreign powers, which should also promote policies to mitigate the detrimental environmental impacts of armed conflicts.

We conclude that armed conflicts remain a poorly understood driver of wildlife population collapses and our results indicate that although individual conflicts can have either positive or negative impacts, the overarching trend is clearly negative and the mere propagation of warzones, regardless of their intensity, is sufficient to heavily deplete wildlife populations. In the interest of preventing wildlife collapses in other parts of the world, we highlight that civil wars can vastly increase the availability of automatic weapons/ammunition which are typically used to deplete wildlife; this consequently leads to intense slaughter and major wildlife declines, especially in more accessible open habitats. This may be easier stated than done, but we conclude that policy strategies that can prevent the consequences of warfare, as shown here, remains a key conservation priority. We realize, however, that this rests on recalcitrant political will to promote robust public policies, which are rare priorities in rebuilding nation-states. It is critical to restore vertebrate community structure, but this may take many decades and require active intervention efforts. A multifaceted strategy to prevent previous war-zones from becoming “empty forests” or “empty savannas’’—severely degrading patterns of diversity, ecosystems functioning and ultimately human welfare—is therefore quintessential.

## Methods

### Study area: landscape and social context

This study was carried out at Quiçama National Park (QNP) and Quiçama Game Reserve (QGR), two contiguous Protected Areas encompassing 960,000 ha and representing Angola’s most important conservation areas^49^. The vegetation structure is subdivided into six units: edaphic communities, open grasslands, tree and clump savanna, wooldland, thicket, and forest^50^.

Approximately 9,000 people live in, but are not legally authorized to occupy, this area. These residents maintain their livelihoods through hunting, fishing, slash-and-burn cassava agriculture, and harvesting of non-timber forest products such as oilseeds and palm fruits^49^. In this study, we selected four villages within both a forest and a savannah landscape (Fig. 1).

Most of the human population is native to the study area, although communities located north of the park also contain residents from Eastern Angola, where armed conflicts were more intense and drove the out-migration of 4 million refugees^44^. On the other hand, QNP and QGR are located in Western Angola and experienced episodic conflicts that were less frequent than those in the east part of the country, ranging from 3 to 15 conflict episodes during the 1975 – 2002 period. All human populations in the study area had easy access to automatic rifles, mainly because of the presence of the military bases that had been installed. Because of the cessation of military conflict in 2002 and subsequent socio-political restoration, most automatic weapons have since been confiscated by the Angolan government^51^. Records of wildlife populations at Quiçama have been reported since the early 1950s with the work of Fernando Frade, by Crawford-Cabral’s in 1968/69^21^ and by Teixeira and Huntley in the 1970s^19^, which produced a mammal checklist. This checklist was recently updated by Braga-Pereira^49^ , Huntley^52^ and Taylor et al.^16^. In early 2000, a rehabilitation project (Operation Noah’s Ark) conducted a species relocation program to the Special Conservation Area, but unfortunately many of these species were exotic to the park^53^. These introduced and reintroduced species are not considered in this study.

### Data acquisition

Countries plagued by war conflicts often experience shortages in funding and human resources, and long-term monitoring programs are virtually non-existent. Human populations that frequently interact with wildlife, mainly through hunting and fishing, develop extensive knowledge about these animals, and can provide detailed information on the hunting history and population abundance of exploited vertebrates^54,55^. We therefore used a LEK approach to retrieve past information regarding hunting and wildlife population trends.

All interviews were performed individually from January 2014 to January 2015 and from January to April 2017, and they did not require local translators as both the interviewer (FB-P) and the informants were fluent in Portuguese. A previous personal contact between FB-P and key informants established a relationship of trust that reduced potential problems such as the researcher been perceived as a ranger and testimonial redundancy, and ensured that all experienced hunters were selected. Even so, of the 118 interviews conducted, it was necessary to exclude three of them from the analyses because there were obvious deviations from other responses. We short-listed local informants who fulfilled the following criteria: He or she (1) was an expert hunter, and (2) lived in one of the eight selected communities prior to or during the civil war. Criterion (2) was included to restrict our sample informants to those that had been hunting in our study area for at least 2 of the 3 time intervals we assessed (pre-, during, and post-war periods). We did not restrict our informants to hunters who began hunting prior to the civil war years (51 years or older) or those who were still hunting (usually 60 years or younger) because otherwise we would fail to obtain information from some hunters who are highly familiar with the local fauna. However, interviewees provided information for the periods in which they were involved in hunting activities. Data compiled here included interviews conducted with 115 experienced local hunters (113 men and 2 women), who were selected using a snowball sampling technique^56^, in which experts indicate another, and so forth. Selected informants ranged in age from 20 to 80 years-old and some hunters were also engaged as farmers, fishers, teachers or community leaders.

Data were collected through individual semi-structured interviews with an illustrated checklist, which provided visual stimulation with photos of all mammal species > 1 kg occurring in the study area. For each game species reported as hunted, we asked about: (i) the main consequences related to the civil war on hunting activity; (ii) an estimate of the perceived relative abundance, which was presented a graphic abundance scale ranging from 0 (when the species population was “absent”) to 4 (when the species was “highly” abundant) (Supplementary Information, Fig. A3). We then asked about the abundance of each species prior to (before 1975), during (from 1975 to 2002) and after the Angolan civil war (2003 to 2017). This perceived abundance was indicated into these three periods to examine any variation over time, and whether this abundance rank increased or decreased during and after the war; (iii) indicate which set of species were hunted during the early (1975 to 1988), at the end (1989 to 2002) and after the war (2003 to 2017), and which species the informant thought would be hunted as a target in the future (after 2018). We also asked about the early and late periods of the civil war to examine whether there was a change in game species pursued during the conflict years. Opportunistic tracks, dung piles, carcasses and direct observations of wildlife were recorded.

### Species data

Species life history: We used the PanTHERIA database^57^ to obtain information on body mass and both the ‘‘PanTHERIA’’ and ‘‘An age’’ databases^58^ to compile information on species fecundity. Annual fecundity (young female per adult female per year) was calculated as (litter size × number of litters per year)/2, assuming a 50:50 sex ratio at birth^59^. We define herbivores, omnivores, and carnivores as “large” if their mean adult body mass exceeded 100 kg, 70 kg, and 22 kg, respectively^60,61^. Medium-sized herbivores, omnivores and carnivores are defined as those with a mean body mass of 5–100 kg, 5–70 kg, and 5–22 kg, respectively. For all mammal orders, we consider small-bodied species as those smaller than 5 kg^62^. Red-listed species are based on the most recent conservation status according to the International Union for the Conservation of Nature^63^.

### Wildlife abundance responses

To perform our models we used as response variables: (i) Delta Abundance (∆_ab_), defined as the species-specific difference in perceived abundance between the pre- and post-war periods. The most depleted species therefore exhibited the highest ∆_ab_ values and those that showed unaltered abundance had ∆_ab_ ≈ 0; (ii) Target species; for each assessed period we specified which potential game species were either a hunting target (1) or not (0).

### Explanatory variables

Our explanatory variables comprised (i) Habitat type: forest or savannah; (ii) Body mass (kg, log_10_); (iii) Informant identity; (iv) Reported relative abundance, which was used as an explanatory variable only for the target species models; and (iv) Target species identity, which was used as an explanatory variable only in the abundance models.

### Qualitative results

The opinions of the interviewees on hunting before, during and after the war periods, reasons for shifts in selectivity of target species, perceived causes of species declines, and what should be done about wildlife declines were noted during the interview and analysed through a ‘’Discourse of the collective subject’’ technique^64^.

### Data analysis

We performed generalized linear mixed models (GLMMs) to examine the effects of each predictor variable on pre- to post-war changes in species relative abundance and which species were selected as hunting targets during the pre-war, war and post-war periods. We considered informant identity as a random variable for both models whereas other explanatory variables were considered as fixed effects. Given that ∆_ab_ values consist of count data ranging between 0 and 4, we used a Poisson error structure. Since the target species data during each war period is binary we used a model structure analogous to binomial regression. We combined all possible models, from the constant to the full model, and performed model selection based on the lowest Akaike information criterion, corrected for small sample sizes (AIC_c_). ΔAIC_c_ then represents the difference between each AIC_c_ and the lowest AIC_c_ value of each model, with ΔAIC_c_ < 2 representing the most likely set of parsimonious models. Finally, we applied a model averaging approach, which represents the beta average of all predictors included in the most parsimonious models. Explanatory variables were z-standardized prior to analyses to ensure comparisons among effect sizes (Supplementary Information, Table A1). All assumptions were examined prior to analyses according to^65^. All inferential analyses were performed in R ver. 3.5.3 (R Core Team 2019) based on the *vegan*^66^ and *rms*^67^ packages.

### Ethics statement

Once this research has the involvement of humans to perform the interviews, we conducted our study following the rules and guidelines of the National Health Council (Resolution 466/12), through the Research Ethics Committee (CEP) of the Universidade Federal da Paraíba (institution to which F.B-P is linked), which approved the execution of the research (license number 59846816.3.0000.5188). The research is registered in the Plataforma Brasil, which is a national and unified database of research records involving human participants. Following the rules of this ethics committee, before beginning an interview, an informed consent form was given to the informant, stating the purpose of the interview and the informant's secrecy, so after having the informed consent from signed, the interview was started. During the fieldwork, the research was initially presented to the leadership of the local community and then the community people were called to a collective meeting. This ensured that the research was also approved by the locals. Also, this research was approved by the Environmental Ministry of Angola (1INBAC.MINAMB/2014 and 148INBAC.MINAMB/2016) and by the Quiçama Park administration (017 / GAB.ADM.M.Q / 2017). We can confirm that this study was primarily based on non-invasive sampling techniques such as direct interviews with explicitly willing informants. Hence, this study did not involve the handling of any specimen, was not assessed by an animal ethics committee.

## Supplementary information

Supplementary Information

## Data Availability

The datasets generated during and/or analysed during the current study are available from the corresponding author on reasonable request.
