# Supplementary material for: Warfare-induced mammal population declines in Southwestern Africa are mediated by species life history, habitat type and hunter preferences
Source: Sci Rep. 2020 Sep 17;10:15428. doi: 10.1038/s41598-020-71501-0 (PMC7498602; doi:10.1038/s41598-020-71501-0)
Supplement: Supplementary file 1 — Supplementary Information [file 41598_2020_71501_MOESM1_ESM.pdf]

Supplementary Information

Warfare-induced mammal population declines in Southwestern are mediated by species life history, habitat type and hunter preferences

Franciany Braga- Pereira; Carlos A. Peres; João Vitor Campos-Silva; Carmen Van-Dúnem Santos; Rômulo Romeu Nóbrega Alves

Figure A1. Percentage of responses obtained from all local hunters interviewed related to different mechanisms through which the Angolan civil war affected native mammal abundance

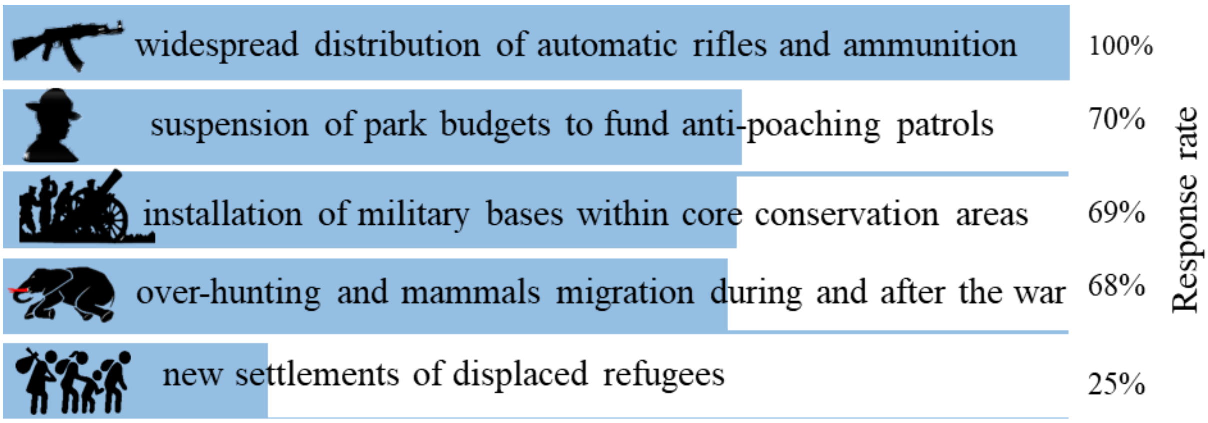

Figure A2. Examples of mammal tracks and visual records obtained during surveys. A: Elephant track. B: Hippopotamus track. C: Red buffalo track. D: Leopard track. E. Common duiker (direct sighting). F: Bushbuck kewel (direct sighting).

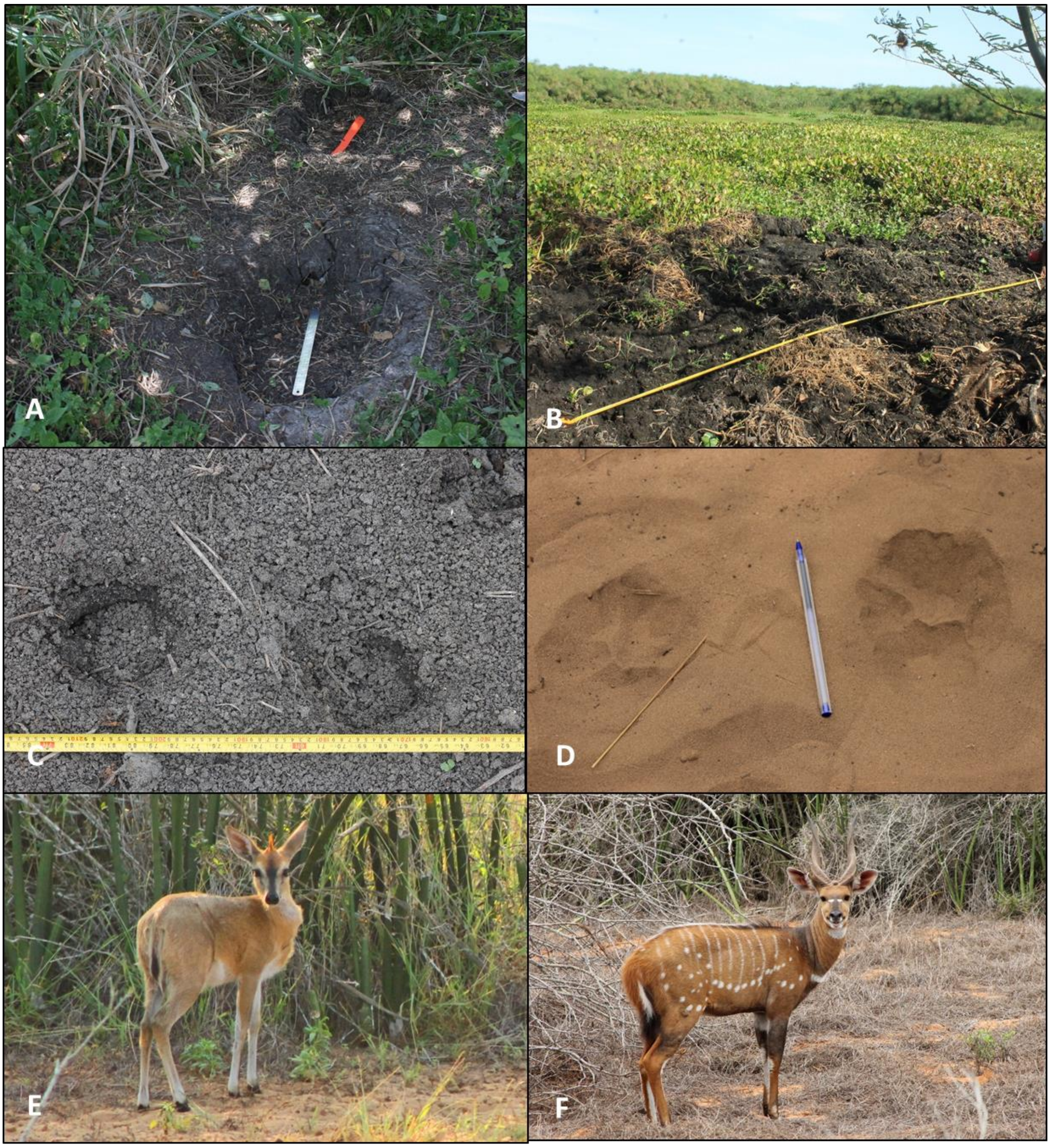

Figure A3. Graphic depiction of the ordinal population abundance scale for different mammal species, consistently used as a visual aid during local interviews, which ranged from 0 (when the local population was conspicuously "absent") to 4 (when the population was "highly" abundant).

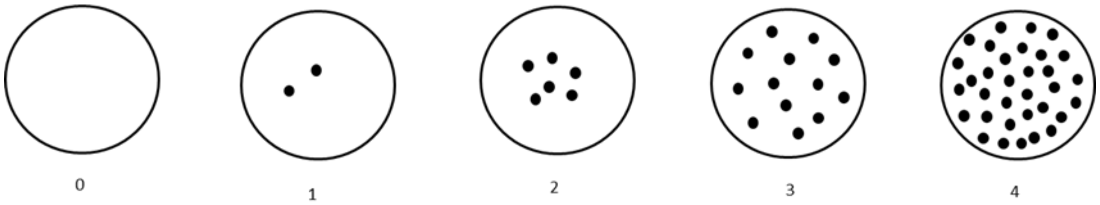
0
1
2
3
4

Table

Table A1. Model average results. Estimated values indicate the coefficient associated with the variable listed on the left. This represents the estimated amount by which the odds (log x) of each response variable would increase if each explanatory variable were one unit higher. Standard errors are an average estimate of how much any given response variable would fluctuate if the study were re-run identically, but with new data. Z-values indicate the degree to which explanatory variables exert a significant effect. Pr(>|z|) are listed as two-tailed p-values that correspond to z-values following a standard normal distribution. Significance levels as following: ns $P > 0.05$; * $P \leq 0.05$; ** $P \leq 0.01$; *** $P \leq 0.001$.

Response variable	Explanatory variable	Estimate	Std. Error	z value	Pr(>|z|)
**Delta abundance**	Habitat	0.34904	0.06908	5.048	0. 0045***
	Body mass	0.22759	0.01207	18.844	<0.0002***
**Target species/ early war years**	Target	0.08622	0.05819	1.48	0.139
	Abundance	-0.798	0.10138	7.865	<2e-16***
	Body mass	1.567221	0.090405	17.321	<2e-16***
	Habitat	0.007543	0.091692	0.082	0.934
**Target species/ late war years**	Habitat	-0.86743	0.13136	-6.604	0. 0401***
	Abundance	0.33118	0.06643	4.986	0. 0618***
	Body mass	0.61054	0.04222	14.462	<0.0002***
**Target species/ post-war**	Abundance	0.52072	0.06473	8.039	<0.0002***
	Habitat	-0.20655	0.14836	1.392	0.164
	Body mass	0.1505	0.03421	4.396	0. 011***

Text S1. Qualitative results in relation to overall hunting pressure at Quiçama during the intermittent cease fire intervals between the beginning and the end of the 27-yr war period. We also include reasons for shifts in abundance of game mammal species, causes of species declines, and suggest remediation efforts.

Fire-weapon disturbance:

When we mentioned the overall impact of the civil war on wildlife, it is important to emphasise that if disturbance is restricted to only exchange of fire between combatants, but no hunting, the animals may be initially frightened, but once they perceive that they are not threatened, they typically remained where they were. However, if some wild animals were slaughtered, they can perceive the non-human smell of blood, and thereby may move elsewhere.

Wildlife migration:

The pronounced reduction in the local fauna is therefore due not only to the slaughter of local animal populations but also because this affects migration patterns. In particular, large carnivores migrate in search of prey, especially lion, which feeds on large prey. The animals that first migrated from open savannahs to forest areas of southern Quiçama and more distant regions were elephants and buffaloes, but those from the forest in northern Quiçama later left the study area, and some began to attack humans, thereby making it riskier to hunt them.

Refugees:

When talking about the impact of civil war on the environment, it is important to consider a wide spatial scale because while, on the one hand, there was out-migration of refugees and soldiers focused on killing residents in some areas, on the other hand areas of low confrontation received those refugees, who contributed to a significant mortality in the local fauna.

Weapons:

In addition to military personnel and local residents who worked in local civil defence, any civilian could barter weapons and ammunition with militants in exchange for natural resources. As weapons and ammunition became cheap, hunters would indulge in much less selective shooting. Also, given their lack of knowledge of the proper use

of personal artillery, and since automatic rifles are not the most suitable weapons for game hunting, many animals were lethally injured but were not subsequently retrieved, thereby greatly contributing to incidental mortality and animal carcasses that were presumably consumed by scavengers. The most common weapons used were AK-47, G3 and mouser, but heavy weapons (e.g.RPG7) were also used to kill elephants.

Local demographics induced by the war:

During the 1980s, refugees and hunters from the Angolan capital city, who would hunt from vehicles and helicopters, invaded the park in large numbers. Their hunting activities, in addition to those of local residents and soldiers based in fixed and mobile military units installed within the park, considerably increasing hunting mortality within the park. The motivation for hunting consisted mainly in acquisition of wild meat for both local consumption and trade, including gourmet commerce. Elephants were slaughtered for the ivory trade, which often involved an exchange of ivory for weapons and other military artillery. Some military personnel and politicians from other countries who visited Angola also hunted for sport or the wildlife trade outside the zones of military conflict (or during months without confrontation) to demonstrate their power. Those travelling in vehicles hunted from the northern to the southern portions of the park through the savannah area. Sometimes the military also hired residents to hunt for them. There were temporary hires of resident hunters who supplied the mobile military bases, which moved throughout the area, as well as long-term hires for supply the fixed military bases. Some soldiers also became established hunters at Quiçama after their demobilisation. However, many local military staff stationed at Quiçama during the war also frequently hunted during their days off to supply the wildlife trade.

Little peace:

In the 1990s it was still possible to find a few herds of red buffalo with about 70 animals. However, after the 1992 ceasefire, access to automatic weapons became even easier, and hunting pressure intensified, so buffalo herds were reduced to 20 individuals each. Currently, buffaloes are either solitary or live in small groups in Central and South of Quiçama. During the years of conflict, hunting was carried out

closer to residential areas, but during the ceasefires and the post-war period (mainly after land-mines were disabled) the area became safer and hunters began to hunt farther away.

Current situation and future prospects:

Hunting activities are still intense today at Quiçama, and in recent years, due to the economic crisis, it has further increased. Activities such as community-based game management could ensure wildlife population recovery as well as bring economic and social benefits to the local population. Also, the ecotourism potential of Quiçama remains very high but unexploited. Its proximity to the capital city of Luanda and highly photogenic and diverse habitats are ideal criteria for a successful ecotourism experience. Educational and health investments are also important to enhance local welfare and quality of life. Without these basic requirements, it remains an enormous challenge to even consider the possibility of sustainable game hunting.
